# Two-dimensional analysis of plasma-derived extracellular vesicles to determine the HER2 status in breast cancer patients

**DOI:** 10.1186/s13058-025-02056-z

**Published:** 2025-06-16

**Authors:** Alexis Wilhelm, Charlotte Flynn, Evelyn Hammer, Johannes Roessler, Bernhard Haller, Rudolf Napieralski, Moritz Leuthner, Sanja Tosheska, Kèvin Knoops, Anjusha Mathew, Giuliano Ciarimboli, Jan Kranich, Lavinia Flaskamp, Siobhan King, Heidrun Gevensleben, Quirin Emslander, Anna Pastucha, Mathias Reisbeck, Lukas Rief, Holger Bronger, Tobias Dreyer, Andreas R. Bausch, Andreas Pichlmair, Thomas Brocker, Reinhard Zeidler, Wolfgang Hammerschmidt, Melanie Piedavent-Salomom, Carmen López-Iglesias, Gabrielle Schricker, Oliver Haydn, Marion Kiechle, Sabine Grill, Ron Heeren, Percy A. Knolle, Olaf Wilhelm, Bastian Höchst

**Affiliations:** 1 Klinik Und Poliklinik Für Frauenheilkunde, TUM University Hospital, Technical University Munich (TUM), Munich, Germany; 2 Therawis Diagnostics GmbH, Grillparzerstrasse 14, Munich, 81675 Germany; 3 Institute of Molecular Immunology, TUM University Hospital, Technische Universität München, Ismaningerstr 22, Munich, 81675 Germany; 4 Research Unit Gene Vectors, Helmholtz Centre München, Munich, Germany; 5 Department of Otorhinolaryngology, University Hospital, Ludwig-Maximilians-Universität München, Munich, Germany; 6 Institute for AI and Informatics in Medicine, TUM University Hospital, Technical University Munich (TUM), Munich, Germany; 7 Heinz-Nixdorf-Chair of Biomedical Electronics, School of Computation, Information and Technology & Munich Institute of Biomedical Engineering, Technical University of Munich, TranslaTUM, Munich, Germany; 8 Maastricht MultiModal Molecular Institute, Maastricht University, Maastricht, The Netherlands; 9 Experimental Nephrology, Medical Clinic D, University of Münster, Münster, Germany; 10 Institute for Immunology, Medical Faculty, LMU Munich, Munich, Germany; 11 Oxford Nanoimaging ONI, Oxford, UK; 12 Institute of Pathology, University Hospital Bonn, Bonn, Germany; 13 Institute of Virology, TUM, Munich, Germany; 14 German Center for Infection Research (DZIF), Partner Site Munich, Munich, Germany; 15 Center for Functional Protein Assemblies, TUM, Garching, Germany; 16 Chair of Cellular Biophysics E27, TUM, Garching, Germany; 17 Matter to Life Program, Max Planck School, Munich, Germany; 18 Center for Organoid Systems and Tissue Engineering, TUM, Garching, Germany; 19 Institute of Structural Biology, Helmholtz Munich, Munich, Germany; 20 Max Plank Institute for Molecular Genetic, Berlin, Germany; 21 Institute of Molecular Immunology, TUM University Hospital, Technische Universität München, Ismaningerstr 22, Munich, 81675 Germany; 22 Center for Infection Prevention, TUM, Munich, Germany; 23 German Center for Infection Research, Munich Site, Munich, Germany

## Abstract

Breast cancer, one of the most common cancers in women, is classified by the expression of hormone receptors and the growth factor receptor HER2, which is important for personalised tumour treatment with HER2-targeted therapies. Tumour biopsies are required for histopathological diagnosis of HER2 expression by breast cancer cells but are subject to sampling error. In this study, we present a method for identifying and analysing cancer-derived EVs from plasma for the detection of HER2 expression in breast cancer without the need for additional processing steps. We detected nano-sized particles through an optimised flow cytometry approach that allows for the identification of HER2-expressing EVs and quantification of their HER2 expression levels. In a clinical study of 115 breast cancer patients, this optimised flow cytometric analysis detected a range of 1.3 to 50 × 10^3^ HER2^+^EVs per µl of plasma. The number of HER2^+^EVs did not correlate directly with tumour size, grade, or metastasis. However, computational integration of data from the quantification of HER2^pos^ EVs per µl/plasma and their HER2 expression levels on a single EV basis allowed for the reliable identification of HER2 expression levels in tumours. Our results reveal the potential for analysing cancer-derived EVs from plasma for the diagnosis and personalised therapy in breast cancer patients.

**Supplementary Information:**

The online version contains supplementary material available at 10.1186/s13058-025-02056-z.

## Introduction

Breast cancer was the most common cancer among women in 2022, with more than 2 million new cases and nearly 700,000 cancer-related deaths, making it the leading cause of cancer-related deaths in women [1–4]. Breast cancer is classified into different subtypes based on the expression of hormone receptors and human epidermal growth factor receptor 2 (HER2) [2, 5, 6]. This classification forms the basis for tailored therapies targeting hormone and growth factor receptors [7]. HER2 expression by breast cancer cells in 15–20% of cancer patients prompted the development of HER2-targeted therapies [8–10], which are an important pillar for the treatment of HER2-positive breast cancer patients and significantly improved patient survival [9]. Breast cancer diagnosis and classification require histopathological and molecular analysis of tumour biopsies [7], and biopsies of metastatic lesions are needed to verify HER2 expression in cancer metastasis. During the disease course, the mutational profile of cancer cells and expression of HER2 in cancer cells might change [11], strengthening the need for repeated tumour biopsies. Furthermore, breast cancer establishes metastases in the lung and brain, which are difficult or impossible to reach for a biopsy.

A promising alternative way of obtaining information on the expression of HER2 by cancer cells is the analysis of extracellular vesicles (EVs) circulating in patients’ plasma [12, 13]. Compared with larger (> 1 µm) microvesicles and apoptotic bodies, EVs are nanoparticles with a mean size of 100–200 nm that are continuously secreted by all body cells [14–17]. The expression of molecular markers, such as tetraspanins (CD9, CD63, CD81), is a characteristic hallmark of EVs [18], and the expression of cell-type-specific markers bears the promise of identifying their cell of origin [19]. Furthermore, EVs contain cell-specific miRNAs that can be used to obtain information on their cell of origin [16]. Notwithstanding these promising features of EVs for cancer diagnosis, their identification and characterisation are hindered by the lack of methods to investigate them at the single particle level, which is necessary to exploit the full potential of information carried by EVs. Ultracentrifugation is an efficient method used for the enrichment of EVs [20], but it may cause damage to EVs during the centrifugation process. Moreover, enrichment of EVs released from cells into the cell culture supernatant may yield fairly pure EV preparations, while ultracentrifugation of plasma will also enrich for abundantly present liposomes and protein complexes, e.g., immune complexes. Furthermore, subsequent bulk analysis methods for investigation of such enriched EVs, like Western blot, do not allow for the discrimination of individual EVs and the analysis of their expression levels of HER2. Alternatively, ultrastructural analysis by immune electron microscopy is highly sensitive, but does not allow for systematic analysis of larger numbers of EVs and is technically demanding. Thus, most current technologies for the analysis of EVs are neither suitable for the detection of single EVs nor for the sensitive and quantitative detection of the expression levels of defined proteins on the surface of single EVs. Thus, we aimed to develop a technology that does not require prior enrichment through ultracentrifugation, allows for a quantitative analysis of single EVs, and sensitive detection of expression levels of defined protein makers on EV surface membranes.

Here, we report that an optimised protocol for flow cytometric analysis enables the quantitative detection of single HER2^+^EVs and quantifies the expression levels of on these EVs released from breast cancer cells in vitro as well as on EVs circulating in the plasma of patients with breast cancer. In 115 breast cancer patients, this optimised flow cytometric detection enabled the characterization of circulating HER2^+^EVs. When combining the results, i.e., the number of circulating HER2^+^EVs per µl plasma and their HER2 expression level, this analysis stratified breast cancer patients for high and low probability of being HER2^+^.

## Results

### Sensitive detection, analysis, and quantification of extracellular vesicles by flow cytometry

**Fig. 1:**
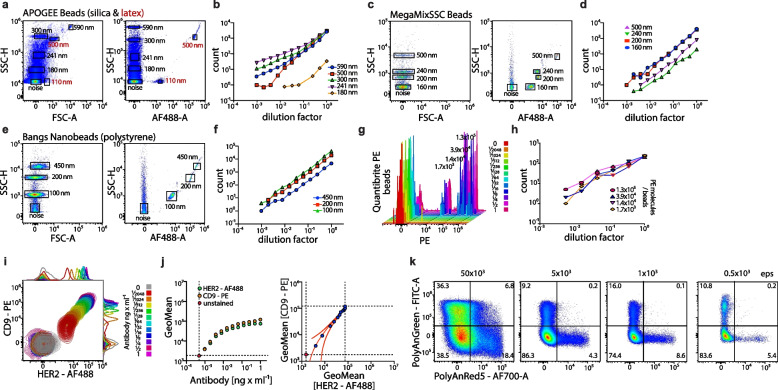
Detection and analysis of nanoparticles by flow cytometry. **a-h** Detection and quantification of different nanoparticles with defined sizes and their fluorescence intensity by flow cytometry; representative plots and quantification of serial dilutions of nanobeads are shown. **i**,**j** Detection of serially diluted anti-CD9-PE- and anti-HER2-AF488-labeled antibodies immobilized on anti-IgG-coated beads by flow cytometry. **k** Flow cytometry-based detection of fluorochrome-labelled polystyrene nanobeads (100 nm) mixed at a 2:1 ratio measured at event rates; representative results are shown

The analysis of single EVs circulating in the blood offers the potential of obtaining information from cancer cells that release them. However, significant challenges must be addressed to enable their reliable detection by flow cytometry. First, EVs have a mean diameter of 100 to 200 nm, which is smaller than the wavelength of light used for their detection. This renders photonic detection difficult. Second, due to their diminutive size, the cumulative surface area of EVs is considerably less than that of cells with a diameter of 10–12 µm by approximately three orders of magnitude. This results in a notable reduction in signals from fluorochrome-labelled antibodies bound to the surface of EVs compared to cells. To investigate whether nanoparticles in the size of EVs can be sensitively detected and analysed by flow cytometry, we used a range of different nanobeads, including nonfluorescent silica beads, fluorescent polystyrene or latex beads of different sizes, and beads with a defined number of fluorescent molecules. Although nanobeads are not an optimal surrogate material for characterising nanoparticles such as EVs by flow cytometry because of a different refractive index [21], they can be employed to ascertain the optimal instrument settings for most sensitive detection. We optimised the flow cytometry measurements to achieve maximal sensitivity for nanoscale particle detection. Using this approach, we reliably detected all nanobeads employed, with diameters ranging from 100 to 590 nm (Fig. 1a,c,e; Suppl. Figure 1a-c). Our flow cytometry-based analysis reliably detected and quantified these nanobeads in serially diluted samples down to a few molecules per µl, demonstrating that sensitive identification and reliable quantification of nanobeads are possible (Fig. 1b,d,f; Suppl. Figure 1a-c). Comparable results were obtained for Quantibrite-beads and nanobeads loaded with different amounts of the fluorescent-labelled antibodies against tetraspanins and HER2 by flow cytometry, which allowed the detection of low concentrations, i.e., 10^–3^ ng/µl of antibodies on nanobeads (Fig. 1i,j; Suppl. Figure 1d-i). Of note, when measuring particle-free ultrapure water, we detected signals that are consistent with electronic background noise from photomultipliers used in flow cytometers to detect fluorescence signals. Furthermore, commercially available buffers used to dilute samples for analysis by flow cytometry may contain contaminating non-fluorescent nanoparticles as well as small air bubbles resulting from sample mixing, which together may be the cause of further background noise signals (Suppl. Figure 1m,n). Therefore, we considered in our analysis only those fluorescence signals detected on nanoparticles that exceed the fluorescence signal from the background noise signals.

In addition to detecting nanoparticles and weak fluorescence signals, flow cytometry-based analysis of EVs must ensure that only single events are analysed. To control for this aspect, we mixed nanobeads labelled either with FITC or with AF700 and determined the conditions that allowed for separate detection and analysis of single fluorochrome-labelled nanobeads. The flow cytometric analysis of nanobeads at event rates of up to 5 × 10^3^ per second resulted in a distinct separation of FITC-labelled from AF700-labelled nanobeads (Fig. 1k). At event rates of 50 × 10^3^ per second and above, however, the separation between the differently labelled nanoparticles was lost (Fig. 1k), indicating that two or more nanobeads were simultaneously analysed. Thus, false positive results may be generated if the event rate during analysis is too high. Together, these experiments demonstrate the potential of flow cytometry to detect and quantify single nanoparticles as small as 100 nm in diameter, as well as for the detection of fluorescence levels on individual nanoparticles.

### Analysis of breast cancer cell-derived EVs by flow cytometry

**Fig. 2:**
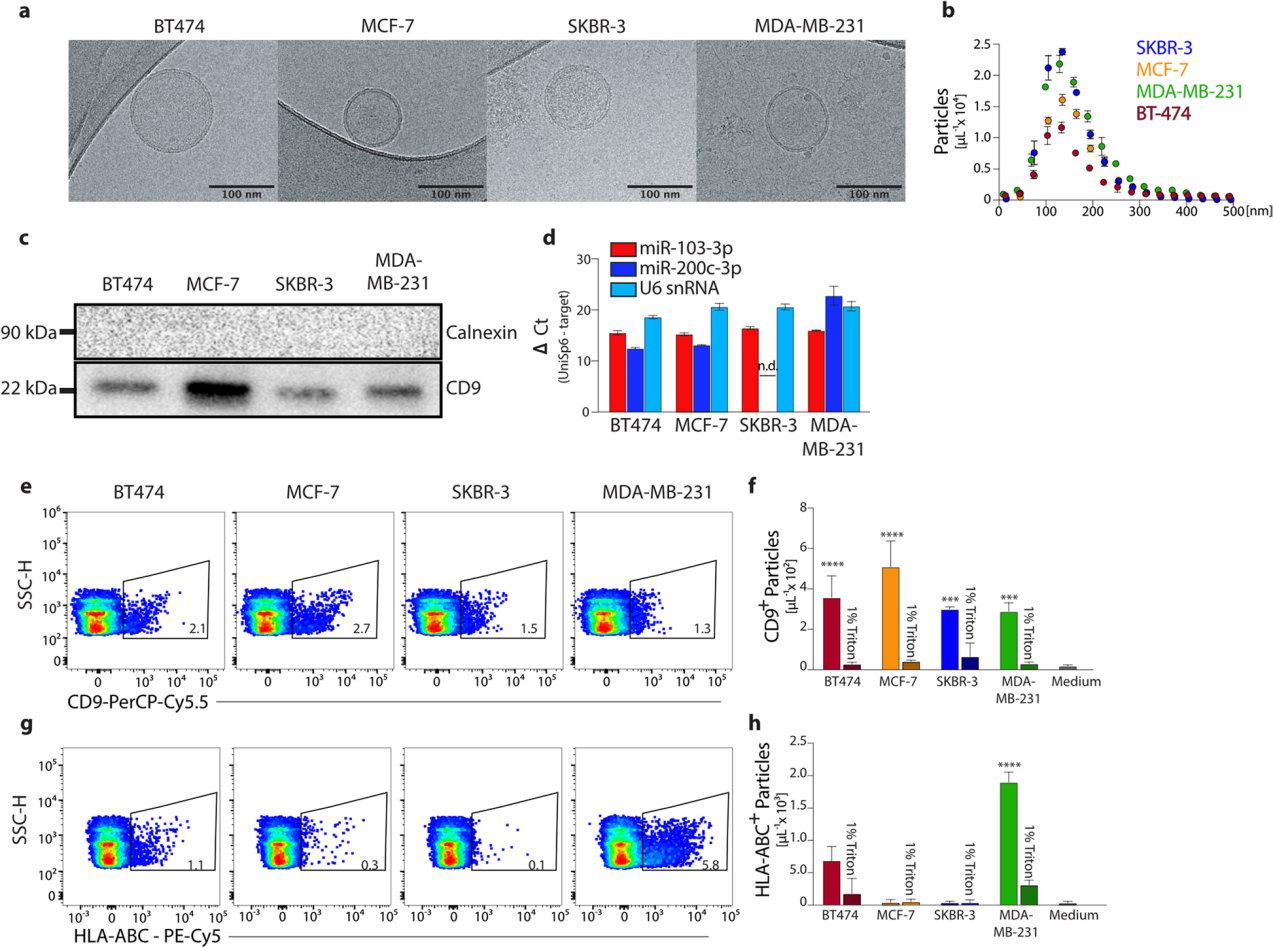
Flow cytometric detection of extracellular vesicles released from breast cancer cells. **a** Representative images of EVs enriched from the supernatants of breast cancer cells from ultrastructural analysis by cryo-electron microscopy (scale bar 100 nm). **b** Nanoparticle tracking analysis of EVs derived from supernatants of breast cancer cells. **c** Western blot analysis of calnexin and CD9 in lysates of EVs from supernatants of different breast cancer cells. **d** Quantitative PCR (∆Ct) of miR-103-3a and miR-200c-3p from lysed EVs by quantitative PCR of triplicate measurements, detection of U6 snRNA as positive control. **e–h** Representative flow cytometric measurements of CD9 and MHC class I expression by EVs from supernatants of different breast cancer cells and quantification; Triton-X-100-mediated destruction of EVs as negative control; statistical significance of differences between Triton-treated and untreated samples calculated by two-way ANOVA; **p < 0.01; ***p < 0.001, **** *p < *0.0001

Having demonstrated that the detection of nanobeads in the size range of EVs is possible by flow cytometry, we employed the optimised settings for characterising EVs released from cancer cell lines. Using different breast cancer cell lines reflecting the different subtypes of breast cancer with distinct expression levels of HER2 that allowed us to stain EVs released from cancer cells for their expression of HER2: BT474 cells (HR^+^HER2^+^, resembling luminal B-like breast cancer), MCF-7 (HR^+^HER2^neg^; resembling luminal A breast cancer), SKBR-3 cells (HR^neg^HER2^+^; HER2-enriched breast cancer) and MDA-MB-231 cells (HR^neg^HER2^neg^; derived from a triple-negative breast cancer). Breast cancer cells were cultured for 24 h in a serum-free medium before supernatants were harvested. First, we performed an ultrastructural analysis by cryo-electron microscopy from samples that were enriched for EVs by ultracentrifugation and revealed that cancer cell-derived EVs displayed the characteristic lipid bilayer membrane and a size of approximately 100—150 nm (Fig. 2a). Nanoparticle tracking analysis (NTA) confirmed the size of breast cancer cell-derived EVs ranging from 50 to 400 nm with a mean diameter of 130 nm (Fig. 2b). We further characterised ultracentrifuged EVs from the different breast cancer cells by Western blot analysis, which confirmed the expression of the tetraspanin CD9 but not of calnexin (Fig. 2c), which are markers of EVs and the endoplasmic reticulum, respectively. EVs may contain cytosolic constituents of the cell they were released from, prompting us to analyse the EVs for the presence of miRNAs reported to be present in the cytosol of breast cancer cells, such as *miR-103-3a* and *miR-200c-3p* [22, 23]. To control for the number of EVs present in the experiments with lysates from EVs, we quantified the U6 snRNA that is considered a stably expressed marker of EVs [24]. We detected *miR-103-3a* in the EVs released from all four breast cancer cell lines, whereas *miR-200c-3p* was detected in all but EVs from SKBR-3 cells (Fig. 2d), which is consistent with the reported absence of miR-200c-3p in SKBR-3 cells [25]. Taken together, these experiments suggest that EVs are released from breast cancer cells into the cell culture supernatant.

We employed our optimised flow cytometry setting to characterise single EVs released from breast cancer cells into the cell culture supernatant without an initial ultracentrifugation step. First, we optimised the concentrations of fluorochrome-labelled antibodies for detecting particular molecules on the surface of EVs by antibody titration to achieve the most sensitive detection by flow cytometry without generating false positive signals from antibody aggregates (Suppl. Figure 2a). Using flow cytometry analysis to detect the exosome marker CD9, we found CD9-expressing EVs in all breast cancer cell lines investigated here (Fig. 2e). Using counting beads for absolute quantification of events, we determined CD9^+^EVs to be present in concentrations ranging between 3 and 5 × 10^3^ per µl in the supernatants of the different breast cancer cell lines (Fig. 2f). The detergent Triton-X-100 was used to dissolve EVs in the cell culture supernatant and led, as expected, to the loss of the CD9 or MHC class I signal detected by flow cytometric analysis (Fig. 2f; Suppl. Figure 2b,c), indicating that the fluorescence signal detected in the supernatants from breast cancer cells was derived from staining of EVs and not from antibody aggregates. Of note, we found CD9 molecules to be expressed on the cell surface and the cytosol of all breast cancer cells from which EVs were analysed (Suppl. Figure 2d), suggesting that EVs reflect the particular characteristics of the cell from which they are secreted.

Next, we addressed whether the differential expression of molecules on the surface of cells is also found on the EVs released from these cells and whether this expression can be detected by flow cytometry. To this end, we stained the four breast cancer cell lines for expression of MHC class I molecules. BT474 and MDA-MB-231 breast cancer cells had high, and MCF-7 cells had low expression levels of MHC class I molecules, both on their cell surface and in the cytosol (Suppl. Figure 2d). SKBR-3 breast cancer cells, however, neither expressed MHC class I molecules on their cell surface nor in the cytosol (suppl. Figure 2e), which is consistent with previous reports on MHC class I expression by these breast cancer cell lines [26]. Notably, by flow cytometric analysis, we detected the expression of MHC class I molecules also on EVs released from BT474 and MDA-MB-231 breast cancer cells but did not detect significant numbers of MHC class I-expressing EVs in the supernatant of MCF-7 and SKBR-3 breast cancer cells (Fig. 2f), indicating that EVs reflected the expression pattern of molecules from the cells they are released from.

### Quantitative flow cytometric detection of HER2^+^EVs released from breast cancer cells

**Fig. 3:**
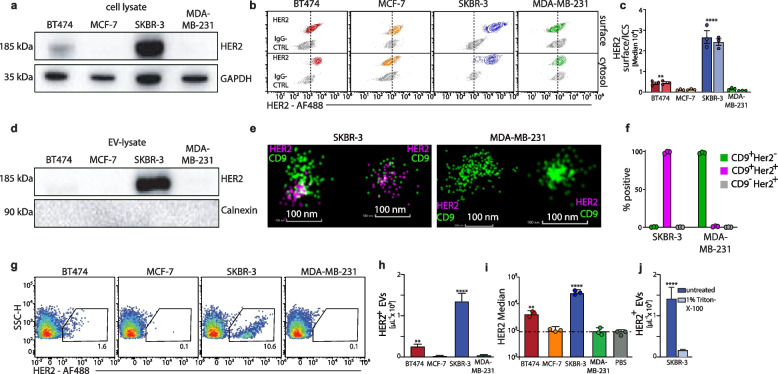
Flow cytometric detection of HER2 expression on EVs from breast cancer cells. **a** Western blot analysis from cell lysates of different breast cancer cell lines for expression of HER2. **b**,**c** Flow cytometric detection of HER2 expression on the cell surface or the cytosol of breast cancer cells and quantification. **d** Western blot analysis from lysates of EVs enriched from supernatants of breast cancer cells for expression of HER2. **e**,**f** Representative dSTORM images of single EVs from cell culture supernatants of SKBR-3 or MDA-MB-231 breast cancer cells stained with anti-CD9-CF488 and anti-HER2-AF647, and quantification; scale bar 100 nm. **g-i** Flow cytometric detection and quantification of HER2^+^EVs in the cell culture supernatants of breast cancer cells and quantification (dotted line = background). **j** Loss of flow cytometric detection of HER2.^+^EVs from the supernatant of SKBR-3 cells after Triton-X-100 treatment. Two-way ANOVA; **p < 0.01; **** *p < *0.0001

The differential expression of HER2 reported for the four breast cancer cell lines provided us with the opportunity to investigate the expression levels of HER2 on EVs released from these breast cancer cells. First, we confirmed the expression levels of HER2 in breast cancer cells using Western blotting of cell lysates, demonstrating that only SKBR-3 and, to a much lesser extent, BT474 cells expressed HER2, whereas MCF-7 and MDA-MB-231 cells did not express HER2 (Fig. 3a). This was confirmed by flow cytometric detection of high expression levels of HER2 on the cell surface as well as in the cytosol of SKBR-3 cells and, to a much lesser extent, of BT474 cells and no expression by MCF-7 and MDA-MB-231 cells (Fig. 3b,c). Western blotting of ultracentrifuged supernatants from breast cancer cells revealed that EVs released from SKBR-3 cells showed a strong signal for HER2. In contrast, in the supernatant from BT474 cells, HER2 was not be detected by western blot (Fig. 3d), although these cells expressed low levels of HER2 (see Fig. 3a). This highlights the challenges associated with conventional bulk exosome analysis methodologies, such as Western blot, to detect low expression levels of proteins. To directly evaluate whether CD9 and HER2 molecules were co-expressed on the same EVs, we performed super-resolution microscopy (dSTORM imaging) of EVs derived from HER2^+^SKBR-3 compared to HER2^neg^MDA-MB-231 breast cancer cells. dSTORM imaging visualised single fluorochrome-labeled antibodies bound on individual EVs and revealed the expression of CD9 molecules on EVs derived from both breast cancer cells. Importantly, only CD9^+^EVs from HER2^+^SKBR3 cells co-expressed HER2 (Fig. 3e,f), consistent with the absence of HER2 expression from MDA-MB-231 cells (see Fig. 3b,c).

Applying the established settings for flow cytometric analysis of nanoparticles, we optimised the use of anti-HER2 antibodies to characterise the breast cancer cell-derived EVs for their expression of HER2 (suppl. Figure 3a). The analysis of the cell culture supernatant without any enrichment methods revealed that HER2 was expressed on EVs released from SKBR-3 and, to a much lesser extent, from BT474 breast cancer cells (Fig. 3g). Determining frequency and expression levels of HER2 on EVs released from confluent breast cancer cells into cell culture supernatants, we found a mean number of 1.4 × 10^3^ HER2^+^EVs per µl and a median HER2-expression level of 3 × 10^4^ fluorescence intensity in the supernatant of SKBR-3 breast cancer cells (Fig. 3h,i, suppl. Figure 3b). The number of HER2^+^EVs in the supernatant of BT474 breast cancer cells was in the range of 2.5 × 10^2^ per µl with a median HER2-expression level of 6 × 10^3^ fluorescence intensity (Fig. 3h,i, suppl. Figure 3b). No HER2^+^EVs were detected in the cell culture supernatants from MCF-7 and MDA-MB-231 breast cancer cells (Fig. 3h,i, suppl. Figure 3b), consistent with the absence of HER2 expression in these cancer cells. Of note, to rule out false-positive events in the flow cytometric evaluation, we treated the supernatant from SKBR-3 breast cancer cells with Triton-X-100 to dissolve EVs. As expected, Triton-X-100 treatment abolished the detection of HER2^+^EVs (Fig. 3j), further supporting the notion that HER2^+^EVs released from breast cancer cells can be detected by flow cytometry.

### Identification of HER2^+^EVs in plasma from breast cancer patients by flow cytometry

After detecting HER2^+^EVs released from breast cancer cells in vitro, we used flow cytometry to analyse EVs in plasma from breast cancer patients (clinical data summarised in Table II) without prior enrichment by ultracentrifugation. Peripheral blood was collected from 23 healthy donors (HDs) and 115 breast cancer patients at the time of cancer diagnosis and before treatment. The patient cohort consisted of 35 patients with HER2^+++^ expression on breast cancer cells defined by immunohistopathology and confirmation for HER2 gene amplification by FISH, 37 patients with intermediate/low HER2^++/+^ expression and no detection of HER2 gene amplification, and 43 HER2^neg^ patients. We incubated plasma samples with optimised concentrations of fluorochrome-labelled anti-CD9 and anti-HER2 antibodies, subsequently diluting the samples 1:1000 to ensure that the critical event rate remained below 5 × 10^3^/second to avoid the generation of false positive results.

**Fig. 4:**
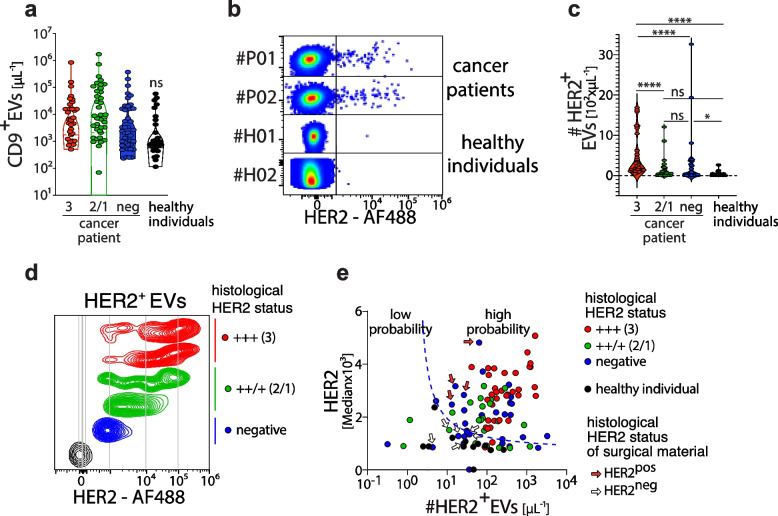
Characterisation of HER2^+^EVs in plasma of breast cancer patients by flow cytometry. **a** Quantification of CD9^+^EVs in the plasma of healthy individuals (*n =* 23) and breast cancer patients (*n =* 115; categorised conducted on their HER2 status). **b** Representative flow cytometric analysis of the HER2 expression by EVs derived from plasma of patients with breast cancer and healthy individuals. **c** Quantification of HER2^+^EVs in plasma of breast cancer patients with different histopathological diagnoses of HER2 expression in cancer tissue (+ +  + (3), +  +/+ (2/1), and negative). **d** Representative flow cytometric analysis of mean fluorescence intensity for HER2 in EVs pregated on HER2 from breast cancer patients with different histological diagnoses of HER2 expression in cancer tissue.** e** Two-dimensional analysis plotting numbers of HER2^+^EVs against HER2-fluorescence levels (median) by EVs stratifying breast cancer patients into two groups with low and high probability of HER2 expression by logistic regression for the count and the median anti-HER2-fluorescence intensity of HER2^+^EVs; red arrow indicates patients initially diagnosed as HER2^neg^ on cancer biopsy who turned out to be HER2^pos^ in surgically removed cancer tissue; unfilled arrow indicates patients diagnosed HER2.^neg^ in surgically removed cancer tissue. Statistical significance of the differences between groups was calculated using the Mann–Whitney test; **p < 0.01; ***p < 0.001, **** *p < *0.0001

CD9^+^EVs were detected in the plasma of all individuals, with no significant difference observed in number between healthy individuals and breast cancer patients (Fig. 4a). The absolute numbers of CD9^+^EVs ranged from 10^3^ to 10^6^ per µl (Fig. 4a). No significant numbers of HER2^+^EVs were identified in the plasma of most healthy individuals (Fig. 4b). The few HER2^+^EVs that were detected in healthy individuals may originate from cells that were physiologically expressing HER2, such as certain epithelial cells from the gastrointestinal tract [27]. In contrast, in plasma from breast cancer patients, we detected a high number of HER2^+^EVs (Fig. 4b; suppl. Figure 4a). To confirm that HER2^+^EVs detected in the plasma of patients by flow cytometry were derived from breast cancer cells, we analysed these EVs for the expression of microRNAs known to be enriched in breast cancer cells [28, 29]. For this, we used anti-HER2-coated beads to enrich circulating HER2^+^EVs from the plasma of patients with HER2^pos^ breast cancer, followed by lysis of EVs and quantitative PCR analysis. We detected *miR-148a-3p* in HER2^+^EVs from the plasma of all three breast cancer patients analysed, but not in EVs isolated from the plasma of healthy individuals (Suppl. Figure 4b). These results suggest that we could detect breast cancer-derived HER2^+^EVs in the plasma of breast cancer patients at the time of diagnosis by flow cytometry.

We wondered whether the absolute numbers of circulating HER2^+^EVs would allow us to distinguish between patients with different histopathologically determined status of HER2 expression in breast cancer tissue. We found significantly higher numbers HER2^+^EVs in the plasma of patients with HER2^+++^ compared to HER2^++^/^+^ expression levels in breast cancer tissue that was determined by histopathological diagnosis (Fig. 4c). Of note, no statistically significant difference was observed between the number of HER2^+^EVs of patients with HER2 ^++^/^+^ expression and healthy volunteers (Fig. 4c). Moreover, no direct correlation was noted for the number of HER2^+^EVs detected by flow cytometry in the plasma of breast cancer patients with the size of the tumour, the tumour grading, or the presence of metastasis (Suppl. Figure 4c-f).

We next addressed the question of whether adding an additional parameter, such as the HER2 expression levels on circulating cancer-derived HER2^+^EVs, which were detected by the fluorochrome-labelled anti-HER2 antibody, might provide further important information. Therefore, we quantified the median anti-HER2 fluorescence intensity detected on HER2^+^EVs (Fig. 4d) and plotted the numbers of HER2^+^EVs against their median expression levels (Fig. 4e, suppl. Figure 4g). To analyse both parameters together, we performed a computational analysis plot was constructed which demonstrated the number of HER2^+^ EVs against the expression level of HER2 per EV. The HER2^+^EVs from healthy individuals (black dots) formed a cluster mainly characterised by low expression intensity of HER2 and was distinctly separated from HER2^+^high EVs and were therefore categorised as low probability (Fig. 4e). The HER2^+^EVs detected in the plasma of patients with documented 3^+^ HER2-expression in cancer tissue by histopathological analysis of biopsy material, however, formed a cluster in this scatter plot analysis, except for two patients (Fig. 4e), consistent with the high HER2 expression levels of the HER2^+^EVs we detected in these cancer patients (red dots). The low number and low expression of HER2 on the EVs of healthy donors, in comparison to the high number and expression of HER2 on the EVs of patients with HER2^high^ tumours, was used to calculate a logistic regression for classification of these two groups. This scatter plot analysis allowed us to separate breast cancer patients with high from those with low probability for being HER2^pos^ (Fig. 4e, suppl. Figure 4g).

Interestingly, we found that the HER2^+^EVs from numerous patients who had no or low (2^+^/1^+^) HER2 expression (green dots) detected by histopathological investigation of cancer biopsies clustered in the same high probability zone for being HER2^pos^ as the HER2^+^EVs from patients with 3^+^ HER2 expression in cancer tissue (Fig. 4e). From four breast cancer patients initially diagnosed as being HER2^neg^, the cancer tissues that were removed during surgery were re-analysed by immunohistochemistry and found to be HER2^pos^. The analysis of the circulating HER2^+^EVs, which were obtained at the time of diagnosis, from these four patients placed these patients into the category with a high probability of being HER2^pos^ (Fig. 4e, red arrows). Likewise, in five patients with no detection of HER2 in cancer biopsy and whose HER2^+^EVs were falling into the low probability zone for being HER2^pos^, the histopathological analysis of surgically removed breast cancer tissue confirmed the absence of HER2 expression in breast cancer (Fig. 4e, white arrows). Taken together, these results indicate that the combined evaluation of the numbers HER2^+^EVs in combination with their HER2 expression level may serve as an indicator for HER expression of breast cancer in patients.

## Discussion

The development of personalised therapies for cancer patients, such as HER2-targeted therapies for patients with HER2^pos^ breast cancer, requires early and reliable identification of patients for such personalised treatment as well as for monitoring during follow-up periods to confirm response to therapy or detect tumour relapse. While the mainstay of cancer diagnosis is the performance of a tumour biopsy and histopathological evaluation of tumour tissue, the tissue sampling from biopsies may not fully represent the diversity of the entire tumour in patients. Furthermore, repeated biopsies in cases of relapsing tumours may not be possible when tumours are too small for detection by current medical imaging technologies and when tumour metastases are located at sites that are difficult to reach by biopsies, such as the lung and brain [30–32]. Thus, analysis of liquid biopsies, i.e., peripheral blood from cancer patients, has emerged as a promising strategy for the molecular analysis of cancer cells in patients, such as the characterisation of cancer-derived extracellular vesicles [13, 33, 34]. Here, we demonstrate that an optimised flow cytometric analysis of EVs from the plasma of breast cancer patients is capable of detecting, quantifying, and characterising single EVs released from breast cancer cells to evaluate the expression of HER2.

The characterisation of EVs is difficult to achieve by flow cytometry due to the nanoscale size of EVs in the range of 100–200 nm, which is below the wavelength of the laser light used for their detection by flow cytometry. Recently, multiplexed analysis of single EVs has been demonstrated to detect biomarker expression on EVs [35]. Our results provide evidence that flow cytometric analysis can be optimised for the detection of cancer-derived single EVs and the analysis of their expression of defined molecular markers, such as HER2. We established a flow cytometric analysis that enabled us to detect and quantify HER2^+^EVs released from breast cancer cell lines and used this system for the detection of breast cancer-derived HER2^+^EVs in the plasma of breast cancer patients. Notably, this optimised flow cytometric analysis did not require enrichment steps such as ultracentrifugation and avoided the problems associated with the bulk analysis of EVs [36], which may overlook EVs with low abundance.

Our data demonstrate that HER2^+^EVs were reliably detected in plasma from patients with breast cancer. Previous studies had identified HER2^+^EVs [37–39], but did not detect a strong correlation of these EVs with the HER2 expression in breast cancer tissue. In line with these studies, the absolute numbers of HER2^+^EVs that we detected in the plasma of breast cancer patients with the optimised flow cytometric analysis also did not correlate with tumour size, tumour stage, or metastasis. Beyond quantifying the number of HER2^+^EVs, the analysis by flow cytometric analysis provides further information on the expression levels of HER2 on single EVs, which may be of interest for the discrimination of high from low to absent HER2 expression in breast cancer cells. Indeed, we found that the combined analysis of the numbers of HER2^+^EVs in plasma together with their HER2 expression levels allowed us to stratify breast cancer patients into a group with a high probability of being HER2^pos^ and one with a low probability. Detection of more than 10^1^ HER2^+^EVs per µl of plasma and mean fluorescence intensity for HER2 surface expression ≥ 1.5 × 10^3^, which is above two standard deviations of the HER2 expression of HER2^+^EVs isolated from healthy individuals, characterised breast cancer patients with a high probability of having an HER2pos tumor. In contrast, detection of less than 10^1^ HER2^+^EVs per µl of plasma and/or HER2 fluorescence intensity ≤ 1.5 × 10^3^ characterised breast cancer patients with a low probability of having an HER2^pos^ tumor. All but two patients with an initial diagnosis of HER2^pos^ breast cancer fell into the high group, indicating that this combined analysis of the numbers of HER2^+^EVs per µl and HER2 expression levels correlates with high HER2 expression in breast cancer tissue. Surprisingly, 13/28 patients with an initial diagnosis of HER^neg^ breast cancer and 11/18 patients with an initial diagnosis of HER2^low^ breast cancer also fell into the group with high probability, suggesting that the analysis of HER2^+^EVs from plasma may provide important information beyond the histopathological analysis of tumour biopsies. When re-investigating some of these breast cancer patients from whom we had immunohistochemical analysis of HER2 expression from the cancer tissue removed during surgery, we found that in four patients who fell into the high probability group but were initially diagnosed as HER2^neg^ from biopsy material, the surgically removed tumours stained positive for HER2. In contrast, in five breast cancer patients who fell into the low probability group and were initially diagnosed as HER2^neg^ from biopsy material, the cancer tissue was also negative for HER2. Together, these results suggest that the combined analysis of the number of HER2^+^EVs and their HER2 expression level correlates with the expression of HER2 in breast cancer tissue. Since a re-evaluation of the HER2 status of surgically removed breast cancer tissue was not available for all patients included in this study, it was not possible to calculate the sensitivity and specificity for the combined analysis of the frequency and HER2 expression level of EVs to correctly detect the HER2 expression of breast cancer tissue. However, the results shown here support the notion that further refinement of the detection of circulating EVs may help in the evaluation of the HER2 status of breast cancer.

Refinement of the flow cytometric analysis for a routine use in future prospective clinical trials will help to evaluate the value of determining both the numbers of HER2^+^EVs and their HER2 expression levels and will demonstrate whether it can provide relevant information on the HER2 status in breast cancer patients in situations where a biopsy may not provide sufficient information, such as in multifocal tumours that may present with different immunohistochemical profiles [7, 40, 41] or in patients with breast cancer metastasis. Moreover, the identification of breast cancer-derived EVs and their isolation may provide the opportunity to identify further molecular markers in the future that may help in the diagnosis and treatment monitoring of breast cancer. However, this methodology is not without limitations. A key drawback of liquid biopsy approaches is the loss of spatial and morphological information typically obtained from tissue biopsies. Furthermore, as the results cannot be normalised to the tumoral input, a negative result can either result from absent HER2 expression or from absent tumoral EVs. Therefore, only positive results can be interpreted.

Taken together, our results demonstrate that the characterisation of plasma-derived HER2^+^EVs in patients with breast cancer by flow cytometric analysis can provide important information on the HER2 expression in breast cancer tissues. This warrants more detailed investigations of plasma-derived HER2^+^EVs in future clinical trials to improve the accuracy of personalised HER2-targeted treatment for breast cancer patients.

## Materials and methods

### Breast cancer cell lines

The following breast cancer cell lines were used: BT474 (HR^+^HER2^+^, resembling luminal B-like breast cancer), MCF-7 (HR^+^HER2^neg^; resembling luminal A breast cancer), SKBR-3 (HR^neg^HER2^+^; resembling HER2-enriched breast cancer) and MDA-MB-231 (HR^neg^HER2^neg^; derived from basal-like/triple-negative breast cancer). BT474, SKBR-3, and MDA-MB-231 breast cancer cells were cultivated in DMEM supplemented with 10% foetal calf serum and 1% penicillin/streptomycin. MCF-7 breast cancer cells were cultivated in RPMI medium supplemented with 10% foetal calf serum and 1% penicillin/streptomycin. The cells were kept in a humidified incubator at 5% CO_2_ and 37 °C. To harvest EVs released into the cell culture supernatant from breast cancer cell lines, cells were cultured in a complete medium until they reached 90–95% confluence. The cells were washed with PBS and cultivated for 24 h in serum-free cell culture medium before the cell culture supernatant was collected. The cells were subsequently centrifuged at 2,000 × g for 15 min to remove large debris and cells and at 10,000 × g for 15 min to remove larger vesicles. The remaining supernatant was then subjected to further analysis.

### Enrichment of EVs from cell culture supernatants of breast cancer cell lines by ultracentrifugation

EV-containing cell culture supernatants were prepared as described above and transferred into ultracentrifugation tubes. EVs were enriched by ultracentrifugation at 100,000 × g for 90 min at 10 °C (Beckman, Germany). The supernatant was carefully discarded, and the EV pellet was resuspended in 50–100 µl of PBS per tube by incubation on ice for one hour with repeated vortexing. The enriched EVs were either directly subjected to further analysis or stored at −80 °C until further analysis.

### Western blot analysis of lysates from breast cancer cells and EVs from the supernatant of breast cancer cells

For Western blot analysis of the cells, the breast cancer cells were detached from the flasks using trypsin, washed twice with PBS, and centrifuged at 2,000 × g for 5 min. The cell pellets were subsequently lysed in RIPA buffer for 30 min. Lysates were centrifuged at 17,000 × g for 15 min to remove cell debris, and the supernatant was stored at−20 °C until further analysis. For Western blot analysis, EVs were pelleted from the supernatants of breast cancer cells by ultracentrifugation as described and resuspended directly in RIPA buffer. After 60 min of incubation with repeated vortexing, the lysed EVs were centrifuged at 17,000 × g for 15 min, and the supernatant was stored at −20 °C until further analysis. After quantification of the protein concentration in the lysates of cells and EVs using the Pierce™ BCA Protein Assay Kit (Thermo Scientific), 30 µg of protein was mixed with Laemmli buffer and denatured at 95 °C for 10 min before being subjected to 10% SDS polyacrylamide gel electrophoresis. The proteins were transferred onto a PVDF membrane by semidry blotting using the Trans-blot Turbo Transfer system (Bio-Rad). The membranes were blocked with 5% dried milk powder dissolved in TBS-T and incubated with primary antibodies against either HER2 (e2-4001; Thermo Fisher, USA; 1/1000), CD9 (Ts9; Invitrogen, USA; 1/1000) or calnexin (AF18, Santa Cruz, USA; 1/1000) overnight. Using an enzyme-labeled secondary antibody (HRP-conjugated goat anti-mouse IgG, polyclonal; Biolegend, USA; 1/20.000), specific proteins were detected with ECL-Select™ Western blotting Detection Reagent (Amersham, USA) and ChemiDoc™ XRS + (Bio-Rad, Germany).

### Nanoparticle tracking analysis of EVs from breast cancer cell supernatants

For nanoparticle tracking analysis (NTA), the cell culture supernatants from breast cancer cells containing EVs were diluted with filtered PBS to a concentration of approximately 1 × 10^4^ per µl before the number and size distribution of EVs were measured by using ZetaView PMX110 (ParticleMetrix, Germany) and ZetaView 8.04.02 software. The pre-acquisition parameters were set to a sensitivity of 75, a shutter speed of 50, a frame rate of 30 frames/s, and a trace length of 15. The post-acquisition parameters were set to a minimum brightness of 20, a minimum size of 5 and a maximum size of 1,000 pixels.

### Nanobeads

To evaluate the impact of the physical size for detecting EVs by flow cytometry, we generated a size scale using commercially available nonfluorescent silica beads or fluorescent polystyrene beads, which have defined sizes. Silica beads: NanoBead Calibration Kit I 0.05–0.1 µm diameter and Kit II 0.2 and 0.5 µm diameter (Bangs Laboratories, USA), ApogeeMix silica/latex beads 110, 180, 241, 300, 500 and 590 nm diameter (APOGEE Flow Systems, UK), Megamix-Plus SSC beads 160, 200, 240 and 500 nm diameter (BioCytex, France), Quantibrite PE Quantification-Beads (BD Bioscience, USA) Ultracomp-Beads (Biolegend, USA) and AlexaFluor-488 or AlexaFluor-700-labeled carboxylated polymethylmethacrylate (PMMA) nanoparticles with 170 nm diameter (PolyAn, Germany). All nanobeads were diluted to a concentration of 10^3^/µl before analysis.

### Cryo-electron microscopy

The samples were added to glow-discharged 200 mesh lacey carbon grids (Agar Scientific, UK) and snap-frozen into liquid ethane after blotting in a Vitrobot (Thermo Fisher Scientific, USA). After clipping, the samples were transferred to a 200 kV Tecnai Arctica transmission electron microscope (Thermo Fisher Scientific, USA) under cryogenic conditions. To locate individual vesicles, whole-grid overviews and detailed mesh overviews were acquired using MAPS software (Thermo Fisher Scientific, USA). Images of the vesicles were then acquired at a nominal magnification of 53.000 × with a Falcon 3EC camera (Thermo Fisher Scientific, USA) in linear mode at a dose of 40 e^−^V^−2^.

### Samples from breast cancer patients at the time of diagnosis

Blood was drawn from healthy donors or breast cancer patients at the time of initial diagnosis of breast cancer and before treatment was started at the Clinic of Obstetrics and Gynaecology, University Hospital München, Klinikum rechts der Isar. In agreement with the ethical votes 107/19S, 2963/10 S, 471/20 S-KH, and 576/19 S all patients provided written informed consent. Blood was drawn from non-fasting healthy donors and breast cancer patients using butterfly and EDTA-coated tubes. The blood samples were centrifuged at room temperature at 2,000 × g for 15 min to separate the plasma from blood cell components. The plasma was centrifuged at 10,000 × g for 15 min at room temperature to remove remaining debris, cells, platelets, and larger vesicles and was frozen in aliquots at −80 °C until analysis.

Tumor biopsie were routinely investigated for the presendce of estrogen receptor (ER), progesterone receptor (PR), and human epidermal growth factor 2 (HER2) expression and categorized as negative if there was a lack or low expression of ER and PR. This was defined by immunohistochemistry staining below 1% for ER and 5% for PR. For HER2, tumor tissue was considered to be negative in the absence or low level HER2 expression (scores 0 and 1 +) or score 2 + in combination with no evidence for HER2 gene amplification by fluorescence in situ hybridizations.

### Isolation of HER2^+^EVs using anti-HER2 antibody beads for quantitative PCR analysis

For the enrichment of HER2^+^EVs for molecular analysis, carboxylated (COOH) particles were labelled with an anti-HER2 antibody. 14.4 × 10^6^ carboxylated particles were activated using 80 µl of EDC/NHS solution and 720 µl of ddH_2_O for 45 min at room temperature under constant agitation. The activated particles were washed three times with 0.5 M MES buffer and mixed 1:2 with anti-HER2 antibody (100 ng/µl), followed by a 1 h incubation at room temperature under constant agitation. Anti-HER2 antibody-labelled particles were incubated with 500 µl samples for two hours at room temperature under constant agitation, followed by two washes with MES buffer. Finally, the particles were blocked with glycine for 30 min at room temperature, washed, and resuspended in PBS under continuous agitation. The anti-HER2 antibody-labelled particles were incubated with plasma from healthy donors or breast cancer patients overnight at 4 °C under constant agitation, washed twice with PBS, and frozen as pellets at −80 °C until further analysis.

To detect miRNA cargo, EVs from plasma or cell culture supernatant were enriched as described above and resuspended in nuclease-free water. The EV content was released by heat denaturation at 95 °C for 10 min. The samples were centrifuged for 15 min at 17,000 × g at 4 °C, and the RNA concentration in the supernatant was determined by Nanodrop analysis and adjusted to 5 ng/µl using nuclease-free water. Reverse transcription was performed using the miRCURY LNA™ RT Kit (Qiagen, Germany) according to the manufacturer´s instructions using the ProFlex™ PCR System (3 × 32-Well; Applied Biosystems). The cDNA was diluted 1:10 with nuclease-free water before being subjected to quantitative real-time PCR using the miRCURY LNA™ SYBR Green PCR Kit (Qiagen, Germany) and primers specific for the detection of *hsa-miR-143-3p*, *hsa-miR-103-3p*, *hsa-miR-200c-3p*, *U6 snRNA* and the *UniSp6* spike-in control. The qPCR and cycling conditions were set up according to the manufacturer´s instructions, the results were analysed using a LightCycler® 480 II (Roche, Germany), and ∆CT values were calculated.

### dSTORM imaging of extracellular vesicles

dSTORM imaging was performed using an EV Profiler Kit (Oxford Nanoimager ONI, UK) according to the manufacturer´s suggestions. Imaging chips were prepared for EV capture by coating the surface with a PS-binding agent for 10 min, followed by 2 × washing. EVs derived from the supernatants of HER2^+^SKBR-3 or HER2^neg^MDA-MB-231 breast cancer cells were enriched by ultracentrifugation to a concentration of 5 × 10^5^ EVs/µl, and 10 µl of solution was added to the coverslip surface to bind for 50 min at room temperature. Unbound EVs were washed before the samples were fixed for 10 min at room temperature. Finally, the samples were washed before 20 µl of dSTORM buffer was added immediately before imaging was performed with the Nanoimager. Super-resolution images were analysed using Collaborative Discovery (CODI) software. The surface was blocked before adding anti-CD9–CF488 and anti-HER2–AF647 in solution for 50 min at room temperature. Before image acquisition on a temperature-controlled Nanoimager S Mark II microscope (ONI, UK), channel mapping was calibrated using 0.1 µm TetraSpeck beads (T7279, Thermo Fisher Scientific). Finally, samples were washed before 20 µl of dSTORM buffer was added immediately before imaging was performed with a Nanoimager (ONI, UK), followed by CODI software analysis.

### Antibodies and staining procedure for EVs

The following antibodies were used: anti-CD9 (1 ng/µl; Clone HI9a; Biolegend, USA), anti-HLA-ABC (1 ng/µl; Clone W6/32; Sony Biotechnology, USA), and anti-CD340 (HER2) – (1 ng/µl: Clone 24D2, Biolegend, USA) AF 488, PE or AF674. All antibodies were titrated to determine the best signal-to-noise ratio for measuring EVs by flow cytometry, as controls PBS and medium were used, or the samples were pretreated with 1% Triton-X-100. Subsequently, the samples were serially diluted, and the results correlated with the dilution. Plasma from healthy donors, breast cancer patients, or cell culture supernatants from breast cancer cell lines was treated for detection of EVs as follows: 10 µl of the sample or control was stained with the optimised concentrations of specific fluorochrome-labelled antibodies and incubated for 30 min at room temperature in the dark. Samples were diluted with PBS 1/100 for cell culture supernatant or 1/1000 for plasma to avoid swarming effects. Absolute counting beads (Thermo Fisher Scientific) were added before flow cytometry analysis to determine the absolute number of events analysed. Flow cytometry data was analysed with FlowJo Software 10.2 (TreeStar, USA).

### Flow cytometry detection of cell surface and intracellular expression of molecules

Breast cancer cells were detached from cell culture flasks using Accutase and washed twice with PBS. For intracellular staining of molecules, cells were incubated in FoxP3 Fixation/Permeabilization Solution (Thermo Fisher, USA) for 60 min at 4 °C. Next, permeabilised cells were washed in permeabilization buffer (Thermo Fisher, USA) and stained with fluorochrome-labelled antibodies against CD9 or HER2 or the respective isotype control antibodies for 60 min at 4 °C in the dark, followed by two washing steps with PBS. For cell surface staining of molecules, breast cancer cells were directly stained with fluorochrome-labelled antibodies against CD9 or HER2 or the respective isotype control antibodies for 60 min at 4 °C in the dark before two washing steps with PBS. Antibody-labelled cells were measured by flow cytometry using an SA3800 Spectral Cell Analyzer (Sony, Germany) in plate loader mode, and the acquired data were analysed using FlowJo Software 10.2 (BD Bioscience, USA).

### Flow cytometry-based analysis of EVs

The samples were analysed on a Sony SA3800 or an ID7000 spectral flow cytometer. The instrument settings are summarised in Table 1. Ten microliters of supernatant from breast cancer cells or platelet-free plasma from breast cancer patients were incubated at 4 °C for 30 min with 1 ng of fluorochrome-labelled antibody per µl sample. After staining, the samples were diluted with NaCl (1:100 for cell culture supernatant; 1:1000 for plasma) and mixed with CountBright™ Absolute Counting Beads (Thermo Fisher, USA) to quantify EVs. Samples were measured using the SA3800 Spectral Cell Analyzer (Sony Biotechnology) in plate loader mode using SSC as a threshold channel. To detect antibody aggregates, separate controls of fluorochrome-labelled antibodies alone were used in parallel. Six hundred microliters of the sample were measured in 9 min. The data were analysed using FlowJo version 10.2 (BD Biosciences, USA).

**Table 1:** Flow cytometer settings used for EV detection. Parameters used for detection of small particles by spectral flow cytometry. Forward scatter (FSC), side scatter (SSC), and a photomultiplier tube (PMT) were used

	**SA3800**	**ID7000**
Flow rate	1	1
Threshold	SSC 0.1%	SSC 4.5%
Gain		
FSC	11%	17
SSC	28%	6.25
V-SSC	n/a	n/a
Fluorescence PMT voltage	68.3%	5.66
Compensation	WLSM	none
Additional settings	No event check	No event check

n/a: could not be set on this device

### Statistics

Statistical analyses were performed with GraphPad Prism 6 (GraphPad Software). Differences between groups were analysed by Student’s two-way unpaired *t*-test or two-way analysis of variance (ANOVA) as indicated. Statistical significance is depicted as follows: *p < 0.05; ** < 0.01; ***p < 0.001; ****p < 0.00001.

Logical regression (counts):

$$P(Y=\text{"}HER2+++\text{"}|\text{log}(Counts))=\text{exp}(\alpha +\beta \text{log}(Counts)\text{log}(Counts))1+\text{exp}(\alpha +\beta \text{log}(Counts)\text{log}(Counts))$$

Logical regression (Median):

$$P(Y=\text{"}+++\text{"}|MedianHER2)=\text{exp}(\alpha +\beta MedianHER2MedianHER2)1+\text{exp}(\alpha +\beta MedianHER2MedianHER2)$$

Combinatation of both models:

$$P\left(Y=\text{+++}|\text{log}\left(Counts\right),MedianHER2\right)=\text{exp}\left(\alpha +\beta \text{log}\left(Counts\right)\text{log}\left(Counts\right)+\beta MedianHER2MedianHER2\right)1+\text{exp}\left(\alpha +\beta \text{log}\left(Counts\right)\text{log}\left(Counts\right)+\beta MedianHER2MedianHER2\right)$$

## Supplementary Information

Supplementary Material 1.

Supplementary Material 2. Table II patient list.

## Data Availability

The data are only available on request due to ethical considerations.
