# Supplementary material for: Two-dimensional analysis of plasma-derived extracellular vesicles to determine the HER2 status in breast cancer patients
Source: Breast Cancer Res. 2025 Jun 16;27:107. doi: 10.1186/s13058-025-02056-z (PMC12168403; doi:10.1186/s13058-025-02056-z)
Supplement: Supplementary file 1 — Supplementary Material 1. [file 13058_2025_2056_MOESM1_ESM.docx]

***Supplementary Figure 1: Detection and analysis of small particles by spectral and conventional flow cytometry.***

***a – d****, Detection of small particles. Different nanobeads with defined sizes and fluorescence intensities were characterized by side scatter high (SSC-H; spectral flow cytometry), forward scatter (FSC), or fluorescence channel detection of AF488. The fluorescence intensity of the different beads analysed via serial dilution is plotted.* ***e - l****, Fluorescence intensity of serially diluted antibodies. anti-HER2–AF488 (****e, f****), anti-CD9–PECy7 (****g, h****), anti-CD9–PE (****I, j****), and anti-CD81–PEDazzle594 (****k, l)*** *antibodies were used. The antibodies were serially diluted to a concentration of 1 ng/µl, followed by the addition of anti-IgG-coated beads (compensation beads). Representative histograms and fluorescence intensities in correlation with dilution are shown.* ***m, n****, Detection of nonspecific background signals. PBS was subjected to sterile filtering through a 0.22 µm filter, removal of air bubbles through a vacuum, and then analysed by spectral flow cytometry, counting beads of 1 µm size were added.*

*** Supplementary Figure 2: Detection of surface markers on EVs and cells by spectral flow cytometry. a****, Western Blot analysis of cell lysate and cell culture supernatant (30µg/lane) for Calnexin and CD9.* ***b****, Titration of anti-CD9-PerCP-Cy5.5 antibody in the cell culture supernatant (SN), NaCl, and DMEM. Plotted is the mean of the CD9-positive particles / µl from three experiments.* ***c,*** *representative pseudocolour dot plots of cell culture supernatant of indicated cell lines or DMEM stained with anti-CD9 or anti-HLA-ABC.* ***D,*** *stained cell culture supernatant (c) treated with triton-X-100.* ***e, f,*** *Representative histograms from intracellular (c) or cell surface staining of BT474, MCF-7, SKBR-3, and MDA-MB-231 breast cancer cell lines with anti-CD9-PE or anti-HLA class I followed by flow cytometry analysis. Isotype control antibodies (light grey) and unstained samples (dark grey) served as negative controls.*

***Supplementary Figure 3.* Titration of HER2 Antibody.**

**a,** *Titration of anti-HER2 – AF488 antibody in the SKBR-3 cell culture supernatant, NaCl, and DMEM. Plotted is the mean of the HER2-positive particles / µl from three experiments.* ***b****, Correlation of the number of HER2^+^ EVs with their fluorescence intensity per EV of the different target lines.*

**

***Supplementary Figure 4. Correlation of HER2^+^ EVs with clinical parameters***

***a****, Representative flow cytometric analysis of the HER2 expression by EVs derived from plasma of patients with breast cancer and healthy individuals treated with Triton-X-100 to dissolve EVs.* ***b,*** *qPCR analysis of hsa-miR-148a-3p expression from lysates of HER2^+^EVs isolated from plasma of breast cancer patients compared to the healthy donor (HD)using anti-HER2 beads.* ***c – f****, analysis of HER2+ EVs in plasma from breast cancer patients subdivides in tumour size (****c****), grading (****d****), metastasis (****e****), or nearby lymph node spread (****f****).* ***g,*** *logical regression model to estimate the probability that a given sample is either Hea or HER2+++.*
