# Supplementary material for: Two-dimensional analysis of plasma-derived extracellular vesicles to determine the HER2 status in breast cancer patients
Source: Breast Cancer Res. 2025 Jun 16;27:107. doi: 10.1186/s13058-025-02056-z (PMC12168403; doi:10.1186/s13058-025-02056-z)
Supplement: Supplementary file 2 — Supplementary Material 2. Table II patient list. [file 13058_2025_2056_MOESM2_ESM.pdf]

Pseudonyme	Breast cancer classification	HER2	Hormone receptor status
B001	iT1c, G3, cN0, iNx	neg	100% /70%
B002	cT2, G3, Ki67 50%, NST, iN+	neg	0% / 0%
B003	cT2, G3, KI67 40%, cN0	neg	0% / 0%
B004	cT2, G2, KI67 10%, cN0	neg	0% / 0%
B005	iT1c, G1, Ki67 2%, iN0	neg	100% / 100%
B006	cT4b, Ki67 5%, iN0, M0	neg	90% / 50%
B006	cT1, G1, Ki67 2%, cN0	neg	95% / 0%
B008	cT3, G2, Ki67 5%, NST, cN0, M0	neg	95% / 95%
B009	Tis, G2, Ki67 30%, NST, Mo	+++	0% / 0%
B010	cT1, G3, Ki67 50%, NST, iN0, M0	+++	0% / 0%
B011	iT2, G2, Ki67 10%, iN+, M1	+++	15% / 0%
B012	pT1c, G3, pN0, M0	+++	75% / 50%
B013	cT2, G2, Ki67 20%, NST, M1	++	95% / 1%
B014	cT2, G2, Ki67 25%, NST, cN0, M0	+++	0% / 0%
B015	cT2, G2, Ki67 25%, NST, cN1, M1	+++	60% / 80%
B016	iT2, G2, NST, iN0	+++	5% / 20%
B017	cT1c, G2, cN1-3, cM0, NST	neg	30%, 0%
B018	cT1, G2, cN1-3, cM0, NST	++	35% / 5%
B019	cT2, G2, cN1-3, cMx, NST	++	20% / 95%
B020	cT4, G2, cN1-3, cM0, NST	++	15% / 90%
B021	cT2, G2, cN1-3, cM0, NST	neg	20% / 95%
B022	cT3, G2, cN1-3, cMo, lobular	neg	60% / 80%
B023	cT1, G2, cN0, cM0, NST	neg	20% / 95%
B024	cT3, G2, cNx, cM0, lobular	neg	25% / 95%
B025	cT2, G2, cN0, cM0, NST	neg	10% / 95%
B026	cT1c, G2, cN1-3, cM0, NST	neg	30% / 90%
B027	cT2, G2, cN0, cM0, NST	neg	20% / 95%
B028	cT2, G3, cN1-3, cM0	neg	40% / 90%
B029	cT3, G2, cN0, cM0, lobular	+	0% / 95%
B030	cT2, G2, cN0, cM0, NST	neg	15% / 95%
B031	cT4b, G2, cN0, cM0, NST	neg	5% / 95%
B032	cT1c, G2, iN0, cM0, NST	neg	35% / 85%
B033	cT2, G2, cN0, cM0, NST	neg	15% / 95%
B034	cT3, G2, cN1-3, cMx, NST	neg	20% / 90%
B035	cT2, G3, cN1-3, cM0, NST	neg	70% / 60%
B036	cT3, G3, cN1-3, cM0, ductal	neg	80% / 70%
B037	cT1, G3, cN1-3, cM0, ductal	+	90% / 80%
B038	cT2, G3, cN0, cM0, ductal	neg	70% / 100%
B039	cT1a, G3, cN0, cM0, ductal	neg	35% / 35%
B040	cT2, G3, cN1-3, cM0, ductal	neg	10% / 99%
B041	cT1c, G3, cN0, cM0, ductal	neg	30% / 90%
B042	cT1c, G2, cN0, cM0, lobular	neg	5% / 80%
B043	cT2, G3, cN0, cM0, ductal	neg	40% / 80%
B044	cT2, G3, cN0, cM0, ductal	neg	35% / 90%
B045	cT1c, G3, cN0, cM0, ductal	neg	25% / 85%
B046	cT2, G3, cN1-3, cM0, NST	neg	30% / 100%
B047	cT2, G3, cN0, cM0, NST	neg	70% / 90%
B048	cT2, G2, cN1-3, cM0, NST	neg	25% / 100%
B049	cT4, G3, cN0, cMx, NST	neg	60% / 100%
B050	cT2, G3, cN0, cM0, NST	neg	25% / 90%
B051	cT4b, G2, cN0, cM0, lobular	neg	5% / 99%
B052	cT1c, G2, cN0, cMc, NST	neg	25% / 0%
B053	cT2, G3, cN0, cM0, ductal	neg	15% / 90%
B054	cT2, G3, cN1-3, cM0, NST	++	85% / 10%
B055	cT1b, G3, cN0, cM0, NST	++	40% / 90%
B056	cT1b, G2, cN0, cM0, NST	++	40% / 90%
B057	cT2, G3, cN1-3, cM0, NST	++	40% / 95%
B058	cT1c, G2, cN0, cM0, NST	++	35% / 100%
B059	cT1c, G2, cN0, cM0, NST	++	40% / 100%
B060	cT4b, G2, cN1-3, cM0, NST	++	30% / 90%
B061	cT1c, G2, cN0, cM0, NST	++	30% / 80%
B062	cT1, G3, cN0, cMx, NST	++	80% / 95&
B063	cT4, G3, cN1-3, cM0, NST	++	25% / 90%
B064	cT1c, G3, cN0, cM0 NST	++	20% / 90%
B065	cT2, G3, cN0, cMx, ductal	++	70% / 80%
B066	cT1, G3, cN0, cM0, ductal	++	30% / 100%
B067	cT1c, G3, cN0, cM0, ductal	++	30% / 95%
B068	cT2, G3, cN0, cM0, ductal	++	70% / 80%
B069	cT2, G3, cN0, cM0, ductal	+	27% / 70%
B070	cT2, G2, cNx, cM0, invasive NST	neg	20% / 90%

B071	cT1c, G2, cN1-3, cM0, invasive NST	neg	10% / 90%
B072	cT2, G2, cNx, cM0, invasive NST	neg	40% / 85%
B073	cT2, G2, cNx, cM0, ductal	neg	20% / 90%
B074	cT2, G2, cN0, cM0, NST	+	25% / 90%
B075	cT1, G3, cN0, cM0	+++	30% / 5%
B076	cT2, G2, cN1, cMx	++	+ / +
B077	cT4, G2, cN1, cM1	++	0% / 0%
B078	cT2, G2, cN0, cM0	++	+ / +
B079	cT2, G2, cN0, cM0	++	+ / -
B080	cT3, G3, cN1, cM0	++	12% / 2%
B081	cT2, G2, cN0, cM1	+++	0% / 0%
B082	cT3, G3, cN1, cM0	+++	1% / 0%
B083	cT1, G3, cN0, cM0	+++	12% / 4%
B084	cT2, G2, cN1, cM0	++	10 % / 0%
B085	cT2, G3, cN0, cM0	+++	+ / +
B086	cT2, cN0, cM0	+++	80% / 0%
B087	cT1, G3, cN0, cM0	+++	0% / 0%
B088	cT2, G2, cN0, cM0	++	+ / 40%
B089	cT1, G2, cN0, cM0	+++	+ / -
B090	cT1, G3, cN0, cM0	++	12% / 12%
B091	cT1, G2, cN1, cM0	+++	0% / 0%
B092	cT1, G2, cN1, cMx	+++	0% / 0%
B093	cT1, G3, cN0, cM0	++	90% / 60%
B094	cT1, G3, cN0, cM0	+++	90% / 80%
B095	cT1, G2, cN0, cM0	+++	0% / 0%
B096	cT2, G3, cN1, cM0	+++	70% / 0%
B097	cT1, G3, cN1, cM0	++	0% / 0%
B098	cT3, G3, cN1, cM0	+++	10% / 30%
B099	cT2, cN0, cM0	+++	90% / 90%
B100	cT2, G2, cN1, cMx	+++	0% / 0%
B101	cT2, G2, cNx, cMx	++	- / -
B102	cT2, G3, cN1, cMx	+++	+ / -
B103	cT2, G2, cN0, cM0	+++	+ / +
B104	cT2, G3, cN1, cM0	+++	+ / 90%
B105	cT3, G1, cN1, cM0	++	- / -
B106	cT4, G2, cN1, cM1	+++	100% / 2%
B107	cT2, G2, cN1, cMx	+++	+ / 90%
B108	cT1, cN0, cM0	++	95% / 0%
B109	cT1, G3, cN0, cM0	+++	+ / +
B110	cT1, G2, cN0, cM0	+++	0% / 0%
B111	cT1, G2, cN0, cM0	+++	95% / 95%
B112	cT2, G2, cN1, cM0	+++	+ / +
B113	cT1, G2, cN1, cM0	+++	0% / 0%
B114	cT1, G, cN1, cM0	+++	+ / +
B115	ct1, G2, cN0, cMx	+++	+ / +
